# ﻿New *Helminthosporium* (Massarinaceae, Dothideomycetes) and *Nigrospora* (Incertae sedis, Sordariomycetes) species associated with walnut (*Juglansregia* L.) in China

**DOI:** 10.3897/mycokeys.109.133431

**Published:** 2024-10-11

**Authors:** Mengting Zou, Fatimah Al-Otibi, Kevin David Hyde, Yong Wang, Xue-Jun Pan

**Affiliations:** 1 Department of Plant Pathology, Agricultural College, Guizhou University, Guiyang, 550025, China; 2 Institute of Plant Health and Medicine, College of Agriculture, Guizhou University, Guiyang Guizhou 550025, China; 3 Department of Botany and Microbiology, College of Science, King Saud University, P.O. Box 22452, Riyadh 11495, Saudi Arabia; 4 Center of Excellence in Fungal Research, Mae Fah Luang University, Chiang Rai 57100, Thailand; 5 Agricultural College, Guizhou University, Guiyang, 550025, China

**Keywords:** Ascomycota, morphology, new taxa, phylogeny, taxonomy

## Abstract

Six collections of ascomycetes were obtained from samples collected from dead branches and leaves of *Juglansregia* in Guizhou and Yunnan provinces, China. By incorporating multigene phylogenetic analysis (ITS, LSU, *rpb2*, SSU, *tef1-α*, *tub2*) supplemented by morphological data, we establish two novel species, namely *Helminthosporiumguizhouense* and *Nigrosporayunnanensis*. In morphology, *H.guizhouense* can be distinguished from *H.caespitosum* by its narrower conidia (13–16 µm vs. 27.3–35.5 µm), and *N.yunnanensis* is characterized by black, globose conidia (16.2 × 14.4 µm). The phylogenetic results further substantiated them as novel taxa. The present study contributes to our comprehension of the range of fungi found in *Juglansregia*, thereby expanding our knowledge of the diversity of fungi within this host.

## ﻿Introduction

Dothideomycetes and Sordariomycetes comprise plant pathogens, endophytes, and saprobes, and they can be identified by their distinct fruiting bodies (Marek et al. 2009; Wijayawardene et al. 2016; Hongsanan et al. 2020; Healy et al. 2022). They are widespread inhabitants of plant tissues including walnut (*Juglansregia* L.). Walnuts are a nutritious and health-beneficial drupaceous nut, globally recognized for their valuable properties (Caglarirmak 2003).

*Helminthosporium* (Massarinaceae, Pleosporales, Dothideomycetes) is a group of asexual Ascomycota proposed by Link (1809) with the type species *H.velutinum*. Most *Helminthosporium* species are saprobes that primarily inhabit various natural substrates, such as plant tissues, wood, bark, dung, and insects, and are also human pathogens (Luttrell 1964, Alcorn 1988; Konta et al. 2023; Hyde et al. 2023, 2024). About 781 taxa have been placed in *Helminthosporium* (http://www.indexfungorum.org, June.2024), but most *Helminthosporium* species differ from the generic type in the development of conidia and conidiophores and therefore are excluded from *Helminthosporium*. Furthermore, very few taxa have molecular data (Hyde et al. 2023). Very few instances of sexual morphs of *Helminthosporium* have been recorded, and the validity of most of these records is questionable as they have not been confirmed by sequence data (Voglmayr and Jaklitsch 2017).

*Nigrospora* (Apiosporaceae, Xylariales, and Sordariomycetes) was proposed by Zimmerman (1902) with the type species *N.panici* (Hyde et al. 2024). Initially, the characterization of *Nigrospora* species relied on morphological features, particularly large dark conidiospores. However, it was discovered that certain key morphological characteristics, such as the size of spores, were similar among species that are actually not closely phylogenetically related (Hao et al. 2020). To accurately identify different species, it is important to use a comprehensive approach that combines both morphological characteristics and phylogenetic analysis (Jayawardena et al. 2020, Maharachchikumbura et al. 2021). Wang et al. (2017) revised the classification of *Nigrospora*, increasing the known species from 15 to 27 by incorporating morphological and molecular data. Chethana et al. (2023) and Hyde et al. (2024) added records of further species. Wang et al. (2017) confirmed the placement of the genus in Apiosporaceae (Xylariales) based on multi-locus molecular phylogeny, including the internal transcribed spacer (ITS), translation elongation factor 1-alpha (*tef1-α*), and b-tubulin (*tub2*) gene regions. This was confirmed by Samarakoon et al. (2021).

Southwest China is a biodiverse region. In this study, six isolates were collected from walnut leaves and dead tissues from Qianxi County, Guizhou Province, and Lincang City, Yunnan Province. This study aimed to determine the taxonomic status of the pathogenic species of walnut in Guizhou and Yunnan provinces through an analysis of both morphological and molecular characteristics. After conducting a multi-locus phylogenetic analysis and morphological examination, two new species, *Helminthosporiumguizhouense*, and *Nigrosporayunnanensis* are identified and introduced.

## ﻿Materials and methods

### ﻿Sample collection, fungal strain isolation, and morphology

Samples exhibiting signs of disease were collected from walnuts in Qianxi County, Guizhou Province, and Lincang City, Yunnan Province, from 2023 to 2024. To establish uncontaminated cultures, disinfection processes were implemented on the sample surfaces (Zhang et al. 2020). Conidia were identified on the surface under a dissecting microscope. These conidia were aseptically extracted from the leaves using a sterilized needle and relocated to a sterile, water-filled drip board. The spores were then dispersed in sterile water, and a small quantity of the resulting spore suspension was absorbed and uniformly dispersed onto a potato dextrose agar (PDA) incorporated with streptomycin. After a 12-hour incubation period at 25 °C, individual germinated spores were selected and transferred to fresh PDA. Additionally, we prepared Malt Extract Agar (MEA) and Oatmeal Agar (OA) media for fungal growth. The cultures were subsequently maintained at room temperature (28 °C) for a duration of 10 days.

VHX-7000 (Keyence, Osaka, Japan), Fully-Integrated Head VHX-7100 (Keyence, Osaka, Japan), and High-Performance Camera VHX-7020 (Keyence, Osaka, Japan) dissecting microscopes were used as vehicles for observing the fungal colonies and fruiting bodies. The morphological characteristics of the fungi were studied and documented using a compound light microscope (Zeiss Scope 5) equipped with an attached camera (AxioCam 208 color). Morphological measurements of the new species’ features were taken using the ZEN 3.0 (blue edition) (Jena, Germany) software. All newly identified taxa have been registered in the Mycobank database (https://www.mycobank.org), accessed on 28 June 2024. For long-term conservation and research purposes, dried holotype specimens were preserved in the Herbarium of the Department of Plant Pathology, Agricultural College, Guizhou University (HGUP). The ex-type cultures have been deposited in the Departmental Culture Collection (GUCC).

### ﻿DNA extraction and sequencing

Upon reaching the border of a 90 mm diameter Petri dish, a sterile scalpel was used to transfer mycelium into a 1.5 mL centrifuge tube for the extraction of genomic DNA. This extraction was performed using PrepMan Ultra Reagent (Applied Biosystems, CA, USA) in line with the manufacturer’s guidelines. The polymerase chain reaction (PCR) amplification was undertaken with a reaction volume of 25 µL. Primer pairs ITS5/ITS4 (White et al. 1990), LR0R/LR5 (Vilgalys and Hester 1990), dRPB2-5F/dRPB2-7cR (Voglmayr et al. 2016), NS1/NS4 (White et al. 1990), Bt2a/Bt2b (Glass and Donaldson 1995), EF1-728F/EF-2 (O’Donnell et al. 1998, Carbone and Kohn 1999) were used to amplify the internal transcribed spacer regions (ITS), partial large subunit nrRNA (LSU) gene, partial DNA-directed RNA polymerase II second largest subunit (*rpb2*), 18S small subunit ribosomal RNA (SSU), partial beta-tubulin (*tub2*) gene, and translation elongation factor 1-alpha (*tef1-α*) gene sequence fragments, respectively.

The PCR thermal cycle program used for amplifying of ITS, LSU, *rpb2*, SSU, *tub2*, and *tef1-α* started with an initial denaturation at 95 °C for 5 minutes. This was followed by 40 cycles of denaturation at 95 °C for 30 s, annealing at 54 °C for 30 s, elongation at 72 °C for one minute each, and a final extension step at 72 °C lasting 10 minutes. Sangon Biotech (Chengdu, China) handled the purification and sequencing of PCR amplicons. Sequences meeting the quality criteria were submitted to GenBank, and their corresponding accession numbers are listed in Table 1, which also contains a complete list of all the strains utilized in this research.

**Table 1.** Species and GenBank accession numbers of DNA sequences used in in the phylogenetic analysis.

Species name	Voucher specimens	GenBank Accession numbers					
		ITS	LSU	*rpb2*	SSU	*tef1-α*	*tub2*
*Byssotheciumcircinans*	CBS675.92	OM337536	GU205217	DQ767646	GU205235	–	–
*Haplohelminthosporiumcalami*	MFLUCC18-0074*	MT928158	MT928156	–	MT928160	–	–
*Helminthosporiellaastilbacea*	COAD2126	MG668862	–	–	–	–	–
*Helminthosporiellastilbacea*	MFLUCC15-0813*	MT928159	MT928157	–	MT928161	–	–
*Helminthosporiellastilbacea*	CPHmZC-01	KX228298	KX228355	–	–	–	–
*Helminthosporiummaquaticum*	MFLUCC15-0357 = S-096*	KU697302	KU697306	–	KU697310	–	–
*Helminthosporiumaustriacum*	CBS139924 = L132*	KY984301	KY984301	KY984365	KY984420	–	–
*Helminthosporiumaustriacum*	CBS14238 = L169	KY984303	KY984303	KY984367	–	–	–
*Helminthosporiumaustriacum*	L137	KY984302	KY984302	KY984366	–	–	–
*Helminthosporiumcaespitosum*	CBS484.77 = L99*	JQ044429	JQ044448	KY984370	KY984421	–	–
*Helminthosporiumcaespitosum*	L141	KY984305	KY984305	KY984368	–	–	–
*Helminthosporiumcaespitosum*	L151	KY984306	KY984306	KY984369	–	–	–
*Helminthosporiumchengduense*	UESTC22.0024 = YQ071048 = CGMCC	ON557751	ON557745	ON563073	ON557757	–	–
*Helminthosporiumchengduense*	UESTC22.0025 = YQ071047	ON557750	ON557744	ON563072	ON557756	–	–
*Helminthosporiumchiangraiense*	MFLUCC21-0087*	MZ538504	MZ538538	–	–	–	–
*Helminthosporiumchlorophorae*	BRIP14521	AF120259	–	–	–	–	–
*Helminthosporiumdalbergiae*	MAFF243853 = H4628 = TS36	LC014555	AB807521	–	AB797231	–	–
*Helminthosporiumendiandrae*	CBS138902 = CPC22194*	KP004450	KP004478	–	–	–	–
*Helminthosporiumerythrinicola*	CPC35291 = CBS145569*	NR_165563	MK876432	MK876486	–	–	–
*Helminthosporiumgenistae*	CBS142597 = L142*	KY984310	KY984310	KY984374	–	–	–
*Helminthosporiumgenistae*	CBS139922 = L129	KY984309	KY984309	KY984373	KY984423	–	–
*Helminthosporiumgenistae*	CBS139921 = L128	KY984308	KY984308	KY984372	KY984422	–	–
***Helminthosporiumguizhouense***	GUCC24-0011*	**PP915799**	**PP949847**	**PP947940**	**PP949912**	–	–
***Helminthosporiumguizhouense***	**GUCC24-0012**	**PP915800**	**PP949848**	**PP947941**	**PP949913**	–	–
***Helminthosporiumguizhouense***	**GUCC24-0013**	**PP915801**	**PP949849**	**PP947942**	**PP949914**	–	–
*Helminthosporiumhispanicum*	CBS136917 = L109*	KY984318	KY984318	KY984381	KY984424	–	–
*Helminthosporiumjuglandinum*	CBS136922 = L118*	KY984321	KY984321	KY984384	–	–	–
*Helminthosporiumjuglandinum*	CBS136911 = L97	KY984322	KY984322	KY984385	KY984425	–	–
*Helminthosporiumjuglandinum*	CBS136912 = L101	KY984319	KY984319	KY984382	–	–	–
*Helminthosporiumjuglandinum*	CBS136913 = L102	KY984320	KY984320	KY984383	–	–	–
*Helminthosporiumleucadendri*	CBS135133 = CPC19345*	KF251150	KF251654	KF252159	–	–	–
*Helminthosporiumlivistonae*	CPC32158 = CBS144413*	NR_160348	NG_064539	–	–	–	–
*Helminthosporiummagnisporum*	MAFF239278 = H4627 = TS33*	AB811452	AB807522	–	AB797232	–	–
*Helminthosporiummassarinum*	CBS139690 = JCM13095 = MAFF239605 = KT1564*	AB809629	AB807524	–	AB797234	–	–
*Helminthosporiummassarinum*	JCM13094 = MAFF239604 = KT838*	AB809628	AB807523	–	AB797233	–	–
*Helminthosporiummicrosorum*	CBS136910 = L96*	KY984329	KY984329	KY984390	KY984427	–	–
*Helminthosporiummicrosorum*	L94	KY984327	KY984327	KY984388	KY984426	–	–
*Helminthosporiummicrosorum*	CBS136916 = L108	KY984323	KY984323	KY984386	–	–	–
*Helminthosporiummicrosorum*	L95	KY984328	KY984328	KY984389	–	–	–
*Helminthosporiumnanjingensis*	HHAUF020380 = ZM020380	KF192322	–	–	–	–	–
*Helminthosporiumoligosporum*	CBS136909 = L93*	KY984333	KY984333	KY984394	–	–	–
*Helminthosporiumoligosporum*	CBS136908 = L92	KY984332	KY984332	KY984393	KY984428	–	–
*Helminthosporiumoligosporum*	L106	KY984330	KY984330	KY984391	–	–	–
*Helminthosporiumquercinum*	CBS136921 = L90*	KY984339	KY984339	KY984400	KY984429	–	–
*Helminthosporiumquercinum*	CBS112393	KY984334	KY984334	KY984395	–	–	–
*Helminthosporiumquercinum*	CBS136915 = L107	KY984336	KY984336	KY984397	–	–	–
*Helminthosporiumsolani*	CBS365.75	KY984341	KY984341	KY984402	KY984430	–	–
*Helminthosporiumsolani*	CBS640.85	KY984342	KY984342	KY984403	–	–	–
*Helminthosporiumsubmersum*	MFLUCC16-1360*	–	MG098787	–	MG098796	–	–
*Helminthosporiumsubmersum*	MFLUCC16-1290PT	MG098780	MG098788	MG098592	MG098797	–	–
*Helminthosporiumsubmersum*	UESTCC22.0021 = Sara08_3 = CGMCC	ON557753	ON557747	ON563075	ON557759	–	–
*Helminthosporiumsyzygii*	CPC35312 = CBS145570*	NR_165564	MK876433	MK876487	–	–	–
*Helminthosporiumtiliae*	CBS136907 = L88*	KY984345	KY984345	KY984406	KY984431	–	–
*Helminthosporiumtiliae*	CBS136906 = L87	KY984344	KY984344	KY984405	–	–	–
*Helminthosporiumtiliae*	L171	KY984343	KY984343	KY984404	–	–	–
*Helminthosporiumvelutinum*	CBS139923 = L131*	KY984352	KY984352	KY984413	KY984432	–	–
*Helminthosporiumvelutinum*	L98	KY984359	KY984359	KY984417	KY984433	–	–
*Helminthosporiumvelutinum*	CBS136924 = L115	KY984347	KY984347	KY984408	–	–	–
*Helminthosporiumvelutinum*	L116	KY984348	KY984348	KY984409	–	–	–
*Helminthosporiumvelutinum*	L117	KY984349	KY984349	KY984410	–	–	–
*Helminthosporiumvelutinum*	UESTCC22.0022 = BY14_2 = CGMCC3.23572	ON557755	ON557749	–	ON557761	–	–
*Helminthosporiumchinense*	UESTCC22.0026 = YQ071,005 = CGMCC3.23570*	ON557754	ON557748	–	ON557760	–	–
*Massarinacisti*	CBS266.62 = JCM14140*	LC014568	AB807539	FJ795464	AB797249	–	–
*Massarinaeburnea*	CBS473.64	OM337528	GU301840	GU371732	GU296170	–	–
*Massarinaeburnea*	CBS139697 = JCM14422 = H3953	LC014569	AB521735	–	AB521718	–	–
*Massarinapandanicola*	MFLUCC17-0596 = KUMCC17-0293*	MG646958	MG646947	–	MG646979	–	–
*Periconiapseudodigitata*	KT1395 = HHUF29370 = CBS139699 = JCM13166 = MAFF239676*	NR_153490	NG_059396	–	NG_064850	–	–
*Pseudodidymosphaeriaspartii*	MFLUCC13-0273	KP325434	KP325436	–	KP325438	–	–
*Pseudodidymosphaeriaspartii*	MFLUCC14-1212	KP325435	KP325437	–	KP325439	–	–
*Pseudosplanchnonemaphorcioides*	L16 = CBS122935	KY984360	KY984360	KY984418	KY984434	–	–
*Pseudosplanchnonemaphorcioides*	MFLUCC13-0533 = CGMCC3.17583	–	KM875454	–	KM875455	–	–
*Pseudosplanchnonemaphorcioides*	MFLUCC13-0611	KP683375	KP683376	–	KP683377	–	–
*Pseudosplanchnonemaphorcioides*	MFLUCC14-0618	KP683372	KP683373	–	KP683374	–	–
*Semiﬁssisporanatalis*	CPC25383 = CBS140659*	KT950846	KT950858	–	–	–	–
*Semiﬁssisporarotundata*	CBS172.93 = CPC549	KT950847	KT950859	–	–	–	–
*Semiﬁssisporatooloomensis*	CBS143431 = CPC31680*	NR_156674	NG_058526	–	–	–	–
*Stagonosporaduoseptata*	CBS135093 = S618*	KF251255	KF251758	KF252260	–	–	–
*Stagonosporaimperaticola*	MFLUCC15-0026 = ICMP21563*	KY706143	KY706133	KY706149	KY706138	–	–
*Stagonosporamultiseptata*	MFLUCC15-0449 = ICMP21562*	NR_165854	NG_068239	–	–	–	–
*Stagonosporapaludosa*	CBS135088*	KF251257	KF251760	KF252262	–	–	–
*Stagonosporaperfecta*	KT1726A = JCM13099 = MAFF239609	AB809642	AB807579	–	AB797289	–	–
*Stagonosporaperfecta*	CBS135099 = S656*	KF251258	KF251761	KF252263	–	–	–
*Stagonosporapseudocaricis*	CBS135132 = S610*	KF251259	KF251763	KF252265	–	–	–
*Stagonosporapseudopaludosa*	CPC22654 = CBS136424*	NR_137840	NG_058052	–	–	–	–
*Stagonosporapseudoperfecta*	CBS120236 = JCM13097 = MAFF239607*	AB809641	AB807577	–	AB797287	–	–
*Stagonosporatainanensis*	KT1866 = MAFF243860	AB809643	AB807580	–	AB797290	–	–
*Stagonosporatrichophoricola*	CBS136764 = D652*	NR_156586	NG_058081	KJ869232	–	–	–
*Stagonosporauniseptata*	CBS135090 = S611*	KF251264	KF251767	KF252269	–	–	–
*Stagonosporauniseptata*	S607 = CPC22151	KF251265	KF251768	KF252270	–	–	–
*Stagonosporauniseptata*	S608 = CPC22150	KF251266	KF251769	KF252271	–	–	–
*Suttonomycesclematidis*	MFLUCC14-0240 = GUCC18	–	KP842917	–	KP842920	–	–
*Suttonomycesrosae*	MFLUCC15-0051*	MG828973	MG829085	–	MG829185	–	–
*Synhelminthosporiumsynnematoferum*	UESTCC22.0023 = HLG072894 = CGMCC3.23574*	ON557752	ON557746	ON563074	ON557758	–	–
*Apiosporamalaysiana*	CBS 102053	KF144896	–	–	–	KF145030	KF144988
*Apiosporapseudoparenchymatica*	LC7234*	KY494743	–	–	–	KY705139	KY705211
*Nigrosporaaurantiaca*	CGMCC 3.18130*	KX986064	–	–	–	KY019295	KY019465
*Nigrosporaaurantiaca*	LC7034	KX986093	–	–	–	KY019394	KY019598
*Nigrosporabambusae*	CGMCC 3.18327*	KY385307	–	–	–	KY385313	KY385319
*Nigrosporabambusae*	LC7245	KY385305	–	–	–	KY385315	KY385321
*Nigrosporabrasiliensis*	CMM 1214*	KY569629	–	–	–	MK753271	MK720816
*Nigrosporabrasiliensis*	CMM 1217	KY569630	–	–	–	MK753272	MK720817
*Nigrosporacamelliae-sinensis*	CGMCC 3.18125*	KX985986	–	–	–	KY019293	KY019460
*Nigrosporachinensis*	LC6851	KX986049	–	–	–	KY019450	KY019579
*Nigrosporachinensis*	CGMCC 3.18127*	KX986023	–	–	–	KY019422	KY019462
*Nigrosporacovidalis*	CGMCC 3.20538*	OK335209	–	–	–	OK431485	OK431479
*Nigrosporacovidalis*	LC158337	OK335210	–	–	–	OK431486	OK431480
*Nigrosporaendophytica*	URM8712 = A.R.M. 687	OM265226	–	–	–	OP572415	OP572418
*Nigrosporaendophytica*	URM8462 = A.R.M. 973*	OM265233	–	–	–	OP572416	OP572420
*Nigrosporafalsivesicularis*	CGMCC 3.19678*	MN215778	–	–	–	MN264017	MN329942
*Nigrosporafalsivesicularis*	LC13553	MN215779	–	–	–	MN264018	MN329943
*Nigrosporaglobospora*	CGMCC 3.20539*	OK335211	–	–	–	OK431487	OK431481
*Nigrosporaglobospora*	LC15839	OK335212	–	–	–	OK431488	OK431482
*Nigrosporagorlenkoana*	CBS 480.73*	KX986048	–	–	–	KY019420	KY019456
*Nigrosporaguangdongensis*	CFCC:53917*	MT017509	–	–	–	MT024493	MT024495
*Nigrosporaguilinensis*	LC7301	KX986063	–	–	–	KY019404	KY019608
*Nigrosporaguilinensis*	CGMCC 3.18124*	KX985983	–	–	–	KY019292	KY019459
*Nigrosporahainanensis*	CGMCC 3.18129*	KX986091	–	–	–	KY019415	KY019464
*Nigrosporahainanensis*	URM8714 = A.R.M.967	OM265228	–	–	–	OM642834	OM793057
*Nigrosporahainanensis*	URM8715 = A.R.M.968	OM265229	–	–	–	OM642835	OM793058
*Nigrosporalacticolonia*	CGMCC 3.18123*	KX985978	–	–	–	KY019291	KY019458
*Nigrosporalacticolonia*	URM8713 = A.R.M. 921	OM265227	–	–	–	OM642833	OM642838
*Nigrosporamagnoliae*	MFLUCC 19–0112*	MW285092	–	–	–	–	MW438334
*Nigrosporamanihoticola*	URM8461 = A.R.M. 645*	OM265224	–	–	–	OM914791	OM869479
*Nigrosporamusae*	CBS 319.34*	KX986076	–	–	–	KY019419	KY019455
*Nigrosporamusae*	LC6385	KX986042	–	–	–	KY019371	KY019567
*Nigrosporaoryzae*	LC2724	KX985959	–	–	–	KY019312	KY019486
*Nigrosporaoryzae*	LC4265	KX985994	–	–	–	KY019335	KY019518
*Nigrosporaosmanthi*	CGMCC 3.18126*	KX986010	–	–	–	KY019421	KY019461
*Nigrosporaosmanthi*	LC4487	KX986017	–	–	–	KY019438	KY019540
*Nigrosporapernambucoensis*	URM8711 = A.R.M.651	OM265225	–	–	–	OM914792	OM869480
*Nigrosporapernambucoensis*	URM8463 = A.R.M. 974*	OM265234	–	–	–	OM914793	OM869481
*Nigrosporaphilosophiae-doctoris*	CGMCC 3.20540*	OK335214	–	–	–	OK431490	OK431484
*Nigrosporapyriformis*	CGMCC 3.18122*	KX985940	–	–	–	KY019290	KY019457
*Nigrosporapyriformis*	URM8716 = A.R.M.970	OM265231	–	–	–	OM513904	OM642839
*Nigrosporarubi*	LC2698*	KX985948	–	–	–	KY019302	KY019475
*Nigrosporasaccharicola*	LC12057	MN215789	–	–	–	MN264028	MN329952
*Nigrosporasaccharicola*	CGMCC 3.19362*	MN215788	–	–	–	MN264027	MN329951
*Nigrosporasacchari-ofcinarum*	CGMCC 3.19335*	MN215791	–	–	–	MN264030	MN329954
*Nigrosporasacchari-ofcinarum*	LC13531	MN215792	–	–	–	MN264031	MN329955
*Nigrosporasingularis*	CGMCC 3.19334*	MN215793	–	–	–	MN264032	MN329956
*Nigrosporasingularis*	LC12068	MN215794	–	–	–	MN264033	MN329957
*Nigrosporasphaerica*	LC2839	KX985964	–	–	–	KY019317	KY019491
*Nigrosporasphaerica*	LC2840	KX985965	–	–	–	KY019318	KY019492
*Nigrospora sp. 1*	LC2725	KX985960	–	–	–	KY019313	KY019487
*Nigrospora sp. 1*	LC4566	KX986022	–	–	–	KY019354	KY019545
*Nigrospora sp. 2*	LC6704	KX986047	–	–	–	KY019373	KY019571
*Nigrosporastoneae*	BRIP 75022a	OR608744	–	–	–	OR604065	OR604067
*Nigrosporavesicularis*	LC0322	KX985939	–	–	–	KY019296	KY019467
*Nigrosporavesicularis*	CGMCC 3.18128*	KX986088	–	–	–	KY019294	KY019463
*Nigrosporavesicularifera*	CGMCC 3.19333*	MN215812	–	–	–	MN264051	MN329975
*Nigrosporavesicularifera*	URM8718 = A.R.M.975	OM265235	–	–	–	OM513905	OM642840
***Nigrosporayunnanensis***	GUCC24-0008*	**PP915796**	–	–	–	**PP947933**	**PP947937**
***Nigrosporayunnanensis***	**GUCC24-0009**	**PP915797**	–	–	–	**PP947934**	**PP947938**
***Nigrosporayunnanensis***	**GUCC24-0010**	**PP915798**	–	–	–	**PP947935**	**PP947939**
*Nigrosporazimmermanii*	CBS 290.62*	KY385309	–	–	–	KY385311	KY385317
*Nigrosporazimmermanii*	CBS 984.69	KY385310	–	–	–	KY385316	KY385322

* = Type specimens. Our strains in this study were in bold.

### ﻿Phylogenetic analyses

**Figure 1.**
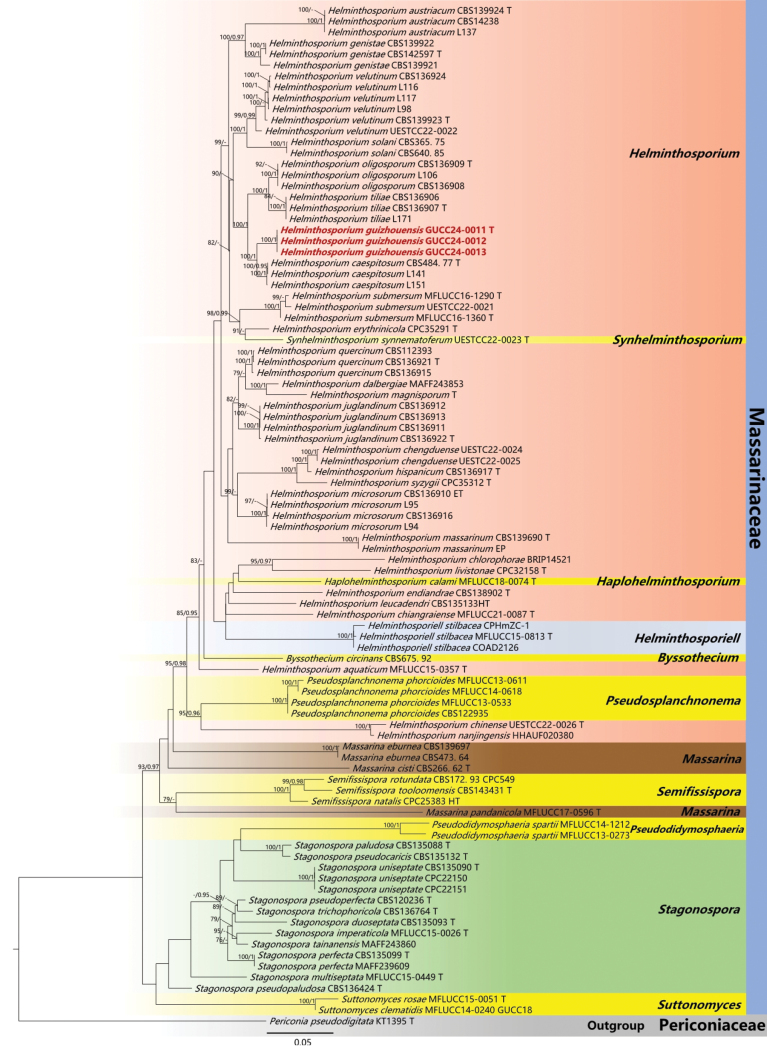
The maximum parsimony tree of 96 *Helminthosporium* taxa is based on ITS, LSU, *rpb2*, and SSU genes. The tree was rooted with *Periconiapseudodigitata* (KT1395). Bootstrap support values for ML greater than 75% and Bayesian posterior probabilities greater than 0.95 are given near nodes, respectively. The new isolates were in red. Ex-type strains were marked by T. The scale bar indicates 0.05 expected changes per site.

**Figure 2.**
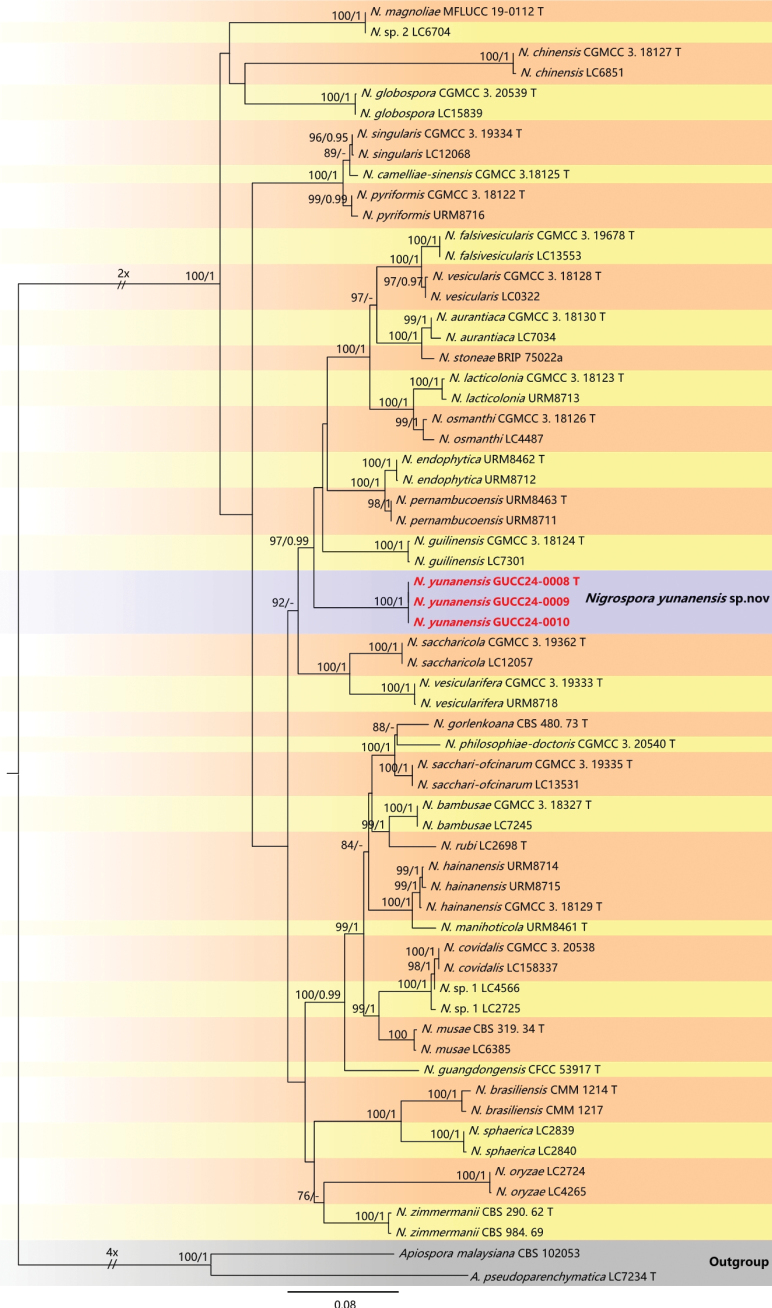
The maximum parsimony tree of 68 *Nigrospora* taxa is based on ITS, *tef1-α*, and *tub2* genes. The tree was rooted with *Apiosporamalaysiana* (CBS 102053) and *A.pseudoparenchymatica* (LC7234). Bootstrap support values for ML greater than 75% and Bayesian posterior probabilities greater than 0.95 are given near nodes, respectively. The new isolates were in red. Ex-type strains were marked by T. The scale bar indicates 0.08 expected changes per site.

Reference sequences obtained from GenBank (Table 1) were utilized to assist with the phylogenetic analyses. Multiple sequence alignments were created using the online platform of MAFFT v.7.307 (http://mafft.cbrc.jp/alignment/server/) (Katoh and Standley 2016). AliView (Larsson 2014) was utilized for manual refinement, with terminal ends and ambiguous regions of the alignment being manually excised. Phylogenetic analyses were performed using concatenated sequences from the six (ITS, LSU, *rpb2*, SSU, *tub2*, and *tef1-α*) through Maximum Likelihood (ML), and Bayesian Inference (BI) methodologies. The Maximum Likelihood analysis was executed on the IQ-TREE web server (http://iqtree.cibiv.univie.ac.at/) (Trifinopoulos et al. 2016). The models is: In Fig. 1, TIM2e+I+G4 for ITS, TIM2e+I+G4 for LSU, TN+F+I+G4 for *rpb2* and K2P+G4 for SSU; In Fig. 2, TNe+I+G4 for ITS, TN+F+I+G4 for *tef1-α*, HKY+F+I+G4 for *tub2*. The number of bootstrap alignments is 1000 (Nguyen et al. 2015). Bayesian analysis (BI) was carried out using PhyloSuite v.1.2.2 as a tool (Zhang et al. 2019). In Fig. 1, SYM+I+G4 as the optimal model for ITS and LSU, HKY+F+I+G4 as the best-fit model for *rpb2*, K2P G4 as the optimal model for SSU; In Fig. 2, SYM+I+G4 as the optimal model for ITS, HKY+F+I+G4 as the best-fit model for *tef1-α* and *tub2*. Four chains were run for 10,000,000 generations and sampled every 500 generations. The initial 25% of the resulting trees were discarded as burn-in, and the remaining trees were used for calculating posterior probabilities in the majority rule consensus tree. The final phylogenetic topology was visualized with FigTree v.1.4.0 (http://tree.bio.ed.ac.uk/software/figtree/) and was modified in Microsoft Office PowerPoint 2019.

## ﻿Results

### ﻿Phylogenetic analyses

For the *Helminthosporium* and related genera (Fig. 1), the phylogenetic trees accommodated 96 sequences listed in Table 1. The strains GUCC24-0011, GUCC24-0012, and GUCC24-0013 were characterized based on their molecular properties, specifically sequencing of the ITS, LSU, *rpb2*, and SSU genes regions. An outgroup consisting of the type strain KT1395 of *Periconiapseudodigitata* was also included in the study based on concatenated datasets, as shown in Table 1. The combined alignment consists of 5596 characters, including ITS (1766 characters), LSU (1648 characters), *rpb2* (1114 characters), and SSU (1065 characters) regions. We constructed two phylogenetic trees: an ML tree and a BI tree. The ML tree was selected to represent the phylogenetic relationship of different *Helminthosporium* taxa (Fig. 1). *Helminthosporiumguizhouense* (GUCC24-0011, GUCC24-0012, and GUCC24-0013) was found to be a sister taxon to *H.caespitosum* (CBS484.77, L141, and L151) with high support values from both ML and BI analyses (ML/BI: 100/1).

For the *Nigrospora* and related genera (Fig. 2), the phylogenetic trees accommodated 68 sequences listed in Table 1. The strains GUCC24-0008, GUCC24-0009, and GUCC24-0010 were characterized based on their molecular properties, and accurate sequencing of the ITS, *tef1-α*, and *tub2* gene regions. *Apiosporamalaysiana* (CBS 102053) and *A.pseudoparenchymatica* (LC7234) were selected as outgroups. The combined alignment consists of 1523 characters, including ITS (571 characters), *tef1-α* (562 characters), *tub2* (385 characters) regions. We constructed two phylogenetic trees: an ML tree and a BI tree. The ML tree was selected to represent the phylogenetic relationship of different *Nigrospora* taxa (Fig. 2). *Nigrosporayunnanensis* (GUCC24-0011, GUCC24-0012, and GUCC24-0013) formed an independent branch without the DNA base differences in three loci supported by strong statistic data (ML/BI: 100/1) and were adjacent to the branch of *N.falsivesicularis* (CGMCC 3.19678 and LC13553), *N.vesicularis* (LC0322 and CGMCC 3.18128), *N.aurantiaca* (CGMCC 3.18130 and LC7034), *N.stoneae* (BRIP 75022a), *N.lacticolonia* (CGMCC 3.18123 and URM8713), *N.osmanthi* (CGMCC 3.18126 and LC4487), *N.endophytica* (URM8712 and URM8462), *N.pernambucoensis* (URM8711 and URM8463), and *N.guilinensis* (LC730 and CGMCC 3.18124) (ML/BI: 97/0.99).

### ﻿Taxonomy

#### ﻿ Helminthosporium guizhouense

Taxon classificationFungiPleosporalesMassarinaceae

M.T. Zou & Yong Wang bis sp. nov.

8CFDB1A2-8D26-5A26-9B65-8FAFD9804B6D

854537

Fig. 3a–r

##### Etymology.

The name refers to Guizhou, the province where the fungus was collected.

##### Diagnosis.

*Helminthosporiumguizhouense* can easily be distinguished from *H.caespitosum* by its narrower conidia (13–16 µm vs. 27.3–35.5 µm).

##### Type.

China • Guizhou Province, QianXi City; 26°56'11.58″N, 105°55'15.46″E; 1235 m; 24 January 2023; from rotten dead branch of *Juglansregia*, coll. M.T. Zou; HGUP24-0007 (holotype); ex-type culture GUCC23-0011 (ITS: PP915799, LSU: PP949847, *rpb2*: PP947940; SSU: PP949912).

##### Description on the natural substrate.

Colonies hairy, brown, or blackish-brown, in groups. Mycelium partly immersed in the substratum, towards the surface forming stroma-like aggregations of light to brown pseudoparenchymatous cells.

##### Culture characteristics.

Colony on PDA 25 mm diam after 2 weeks in an incubator under dark conditions at 28 °C, irregular circular, fat, raised, undulate, rough, with white and denser mycelium at the center, with white to deep-gray to creamy yellow, entire margin; reverse cream to yellow, with dark yellowish-brown spots. Teleomorph: Unknown. Anamorph: Conidiophores macronematous, erect, straight, or slightly curved, cylindrical, smooth, 171–718 μm long, 12–25 μm wide at the base, tapering to 7–13.5 μm near the apex, arising solitary or in fascicles from the stroma cells, erect, simple, straight or flexuous, thick-walled, brown to dark brown, with sympodial proliferation, 1–13-septate. Conidia 61–114 × 13–16 µm (x̄ = 85 × 18, n = 45), gradually tapering to 3–7 μm (x̄ = 5, n = 45) at the distal end, with a 4–10 μm (x̄ = 6, n = 42) wide, blackish-brown to black scars at the base, straight or flexuous, solitary, obclavate to rostrate, smooth-walled, hyaline, pale golden brown to brown, 8–12-distoseptate, with angular lumina; wall up to 6 µm thick.

**Figure 3.**
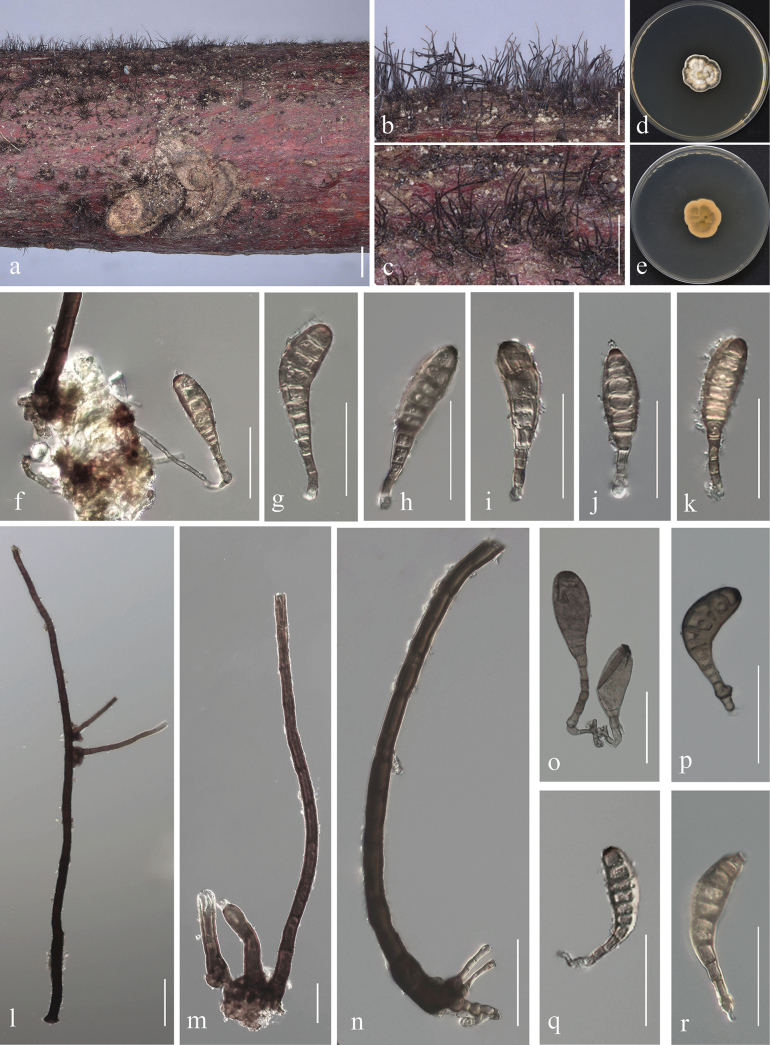
*Helminthosporiumguizhouense* sp. nov. (HGUP24-0008, holotype) on rotten dead branch of *Juglansregia***a–c** colonies on the natural substrat; **d, e** culture on PDA after 2 weeks (**d** above **e** reverse) **f** conidiophore bases, stroma cells, and conidia **l** conidiophore **m** colony, conidiophores, and stroma cells **n** conidiophore **g–k, o–r** conidia. Scale bars: 1000 µm (**a**); 500 µm (**b, c**); 50µm (**f–r**).

##### Habit.

Saprobic on decaying wood of *Juglansregia*.

##### Distribution.

China, Guizhou Province, Qianxi City

##### Other material examined.

China • Guizhou Province, Qianxi City; 105°92'E, 26°93'N; 1235 m; 24 January 2023; from rotten dead branch of *Juglansregia*, coll. M.T. Zou, HGUP24-0008 (holotype); living culture GUCC24-0011, GUCC24-0012, and GUCC24-0013.

##### Notes.

Based on the multi-gene phylogenetic tree (Fig. 1), our strains are clustered in a distinct branch adjacent to the strain of *Helminthosporiumcaespitosum* (CBS 484.77). Topologically, there is a clear genetic distance between these taxa with ML-BS = 100%, BYPP = 1 support. When comparing the ITS, LSU, *rpb2*, and SSU nucleotides of *H.guizhouense* with *H.caespitosum* in the clade, there are 22 bp (0 gap) differences of 569 bp in ITS, 2 bp (0 gap) differences of 904 bp in LSU, and 38 bp (0 gap) differences of 401 bp in rpb2, and 4 bp (0 gap) differences of 1098 bp in SSU. Our collection of *H.guizhouense* (HGUP24-0008) differs significantly from the holotype of *H.caespitosum* (WU 38825 and WU 38826) (Voglmayr and Jaklitsch 2017) in the length of conidiophores (171–718 × 9.5–23 µm vs. 27–37 × 12.2–14.5 µm), the size of conidia (61–114 × 13–16 µm vs. 82–109 × 27.3–35.5 µm), the number of septa (8–12 vs. 6–10) and the wall thickness of angular lumina (6 μm vs. 8 μm). In addition, the colonies on the natural substrate of *H.guizhouense* are hairy, brown, or blackish brown, in groups, whereas *H.caespitosum* is dark-red-brown, scattered, or crowded. Through our analysis and classification process, we have identified these three strains as a new species *Helminthosporiumguizhouense*.

#### ﻿ Nigrospora yunnanensis

Taxon classificationFungiXylarialesApiosporaceae

M.T. Zou & Yong Wang bis sp. nov.

A666839F-A87E-5F0C-BC50-27AE196CA3ED

854538

Fig. 4a–k

##### Etymology.

The name refers to Yunnan, the province where the fungus was collected.

##### Diagnosis.

*Nigrosporayunnanensis* is characterized by black, globose conidia (16.2 × 14.4 µm).

##### Type.

China • Yunnan Province: Lincang City; 23°40'26.08"N, 99°56'47.70″E; 1900 m; 22 Dec 2023; on *Juglansregia*, coll. M.T. Zou; HGUP24-0007 (holotype); ex-type culture GUCC24-0008 (ITS: PP915796, *tef1-α*: PP947933, *tub2*: PP947937).

##### Culture characteristics.

Colonies on PDA reaching 90 mm diam after ten days at 25 °C. The anterior surface and posterior surface are white, while the mycelium is thick and fluffy. Colonies on OA reach 90 mm diam. after ten days at 25 °C. The mycelium is circular, filiform, fluffy, while the surface and reverse are initially white, becoming gray to black, or black and producing a few black areas with age. Colonies on MEA reaching a diameter of 90 mm after ten days at 25 °C. The mycelium circular, filiform, thick, and fluffy. The surface and reverse are initially white, but they become gray to dark black with abundant black areas spreading from the periphery to the center as they age. Sexual morph undetermined. Asexual morph on OA: Hyphae 2.5–8 µm diam, smooth, hyaline to pale brown, branched, septate. Conidiophores smooth, hyaline to brown, branched, septate, sometimes reduced to conidiogenous cells. Conidiogenous cells (n = 30) 8–14 × 6–10 µm (av. = 10.4 × 8.2 µm), aggregated in clusters on hyphae, pale brown, subglobose to ampulliform. Conidia (n = 40) 14.5–18.5 × 11–17.5 µm (av. = 16.2 × 14.4 µm) solitary, globose to subglobose, black, shiny, smooth, aseptate.

##### Habitat.

On *Juglansregia*.

##### Known distribution.

China, Yunnan Province, Lincang city.

##### Additional material examined.

China • Yunnan Province: Lincang city; 23°67'N, 99°94'E; 1900 m; 22 Dec 2023; on *Juglansregia*; coll. M.T. Zou; HGUP24-0007; living culture GUCC24-0008, GUCC24-0009, and GUCC24-0010.

##### Notes.

**Figure 4.**
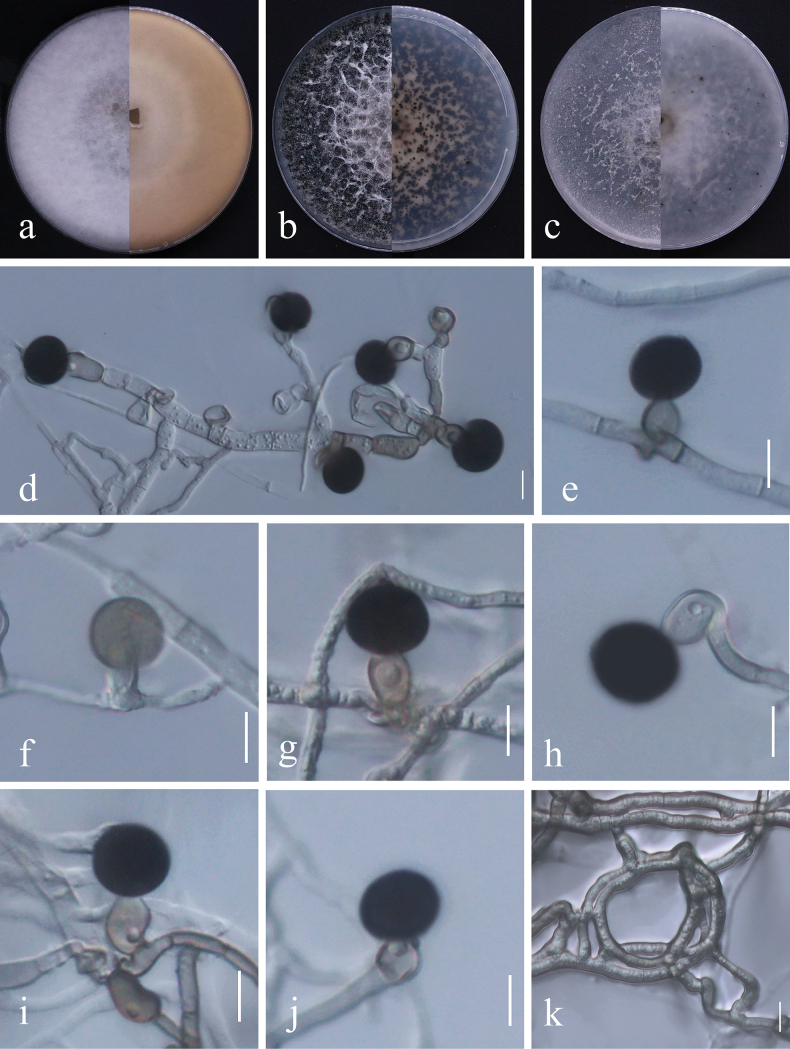
*Nigrosporayunnanensis* (GUCC23-0008) **a**–**c** culture characteristics on media after ten days (**a** on PDA**b** on CMA **c** on OA) **d**–**j** conidia attached to conidiogenous cells **k** coiled hyphae. Scale bars: 10 µm (**d–k**).

Three isolates from walnut leaves were obtained in this study and clustered in a well-supported clade distinguished from other known species (Fig. 4). *Nigrosporayunnanensis* formed an independent branch. Morphological differences (Table 2) support that they belong to different taxa.

**Table 2.** Comparison of conidia and conidiogenous cells of *Nigrospora* species related to this study.

Species	Strain	Conidia (µm)	Conidiogenous cells (µm)	Reference
*Nigrosporaendophytica*	URM8462	10–17.5	6.2–10	(Brito et al. 2023)
*N.guilinensis*	CGMCC 3.18124	11.5–15	6–11 × 4–7.5	(Wang et al. 2017)
*N.pernambucoensis*	URM8463	12.5–20	5–22.5 × 5–12.5	(Brito et al. 2023)
*N.saccharicola*	CGMCC 3.19362	13.5–16.5	7.5–10.5 × 5–7.5	(Raza et al. 2019)
*N.vesicularifera*	CGMCC 3.19333	11–19	7.5–10 × 12.5–15.5	(Raza et al. 2019)
*N.yunnanensis*	GUCC24-0008	14.4 × 16.2	6–10 × 8–14	This study

## ﻿Discussion

In the family Massarinaceae, along with *Helminthosporium*, there are ten other accepted genera: *Byssothecium*, *Haplohelminthosporium*, *Helminthosporiella*, *Massarina*, *Mirohelminthosporium*, *Pseudodidymosphaeria*, *Pseudosplanchnonema*, *Semifissispora*, *Stagonospora*, and *Suttonomyces* (Wijayawardene et al. 2022). *Helminthosporium* is polyphyletic, as confirmed by Konta et al. (2021), and its members were found mixed with other taxa of *Byssothecium*, *Helminthosporiella*, and *Pseudosplanchnonema*. According to a study by Chen et al. (2022), a new genus (*Synhelminthosporium*) was identified through morphological examination and multi-locus phylogenetic analyses. Most *Helminthosporium* species are saprobic, primarily found on woody plant materials. However, some are plant pathogens, and others thrive on fungi, particularly in Diaporthales, but the role of these *Helminthosporium* species on their fungal hosts is still uncertain (Voglmayr and Jaklitsch 2017).

*Nigrospora* belongs to the Apiosporaceae, and are endophytes, saprobes, and plant pathogens, causing harm to economically important plant species within both forestry and agricultural domains. Examples include *N.oryzae* causing panicle branch rot disease on *Oryzasativa* in China (Liu et al. 2021), *N.sphaerica* causing Leaf blight disease of Cacao in the Philippines (Villanueva et al. 2023), *N.chinensis* causing stem spot on dragon fruit in China (Guo et al. 2024). *Nigrospora* is also a human pathogen. *N.oryzae* and *N.sphaerica* can cause human corneal keratitis (Ananya et al. 2014; Takayama et al. 2024). In addition, *N.yunnanensis* was isolated from *Juglansregia*, which could potentially be a pathogen for the walnuts.

The nutritional benefits of walnut kernels are substantial, as they are rich in fat, protein, vitamins, and minerals, while also containing essential compounds such as flavonoids and phenolic acids (Caglarirmak 2003). Moreover, walnuts are hosts to multiple forms of microfungi, including pathogens, endophytes, and saprobes (Zhang et al. 2024). Given this, it is crucial to undertake a comprehensive study of the microfungi present on walnuts in previously unexplored regions, for instance, the provinces of Yunnan and Guizhou in China, and to perform a thorough taxonomic classification of these microorganisms.

## Supplementary Material

XML Treatment for Helminthosporium guizhouense

XML Treatment for Nigrospora yunnanensis

## ﻿Additional information

### Author ORCIDs

Fatimah Al-Otibi https://orcid.org/0000-0003-3629-5755

Kevin David Hyde https://orcid.org/0000-0002-2191-0762

### Data availability

All of the data that support the findings of this study are available in the main text.
